# Stillbirths in urban Guinea-Bissau: A hospital- and community-based study

**DOI:** 10.1371/journal.pone.0197680

**Published:** 2018-05-23

**Authors:** Morten Bjerregaard-Andersen, Najaaraq Lund, Anne Sofie Pinstrup Joergensen, Frida Starup Jepsen, Holger Werner Unger, Mama Mane, Amabelia Rodrigues, Staffan Bergström, Christine Stabell Benn

**Affiliations:** 1 Bandim Health Project, INDEPTH Network, Apartado 861, 1004 Bissau Codex, Guinea-Bissau; 2 Research Center for Vitamins and Vaccines (CVIVA), Statens Serum Institute, Copenhagen, Denmark; 3 Department of Endocrinology, Hospital of Southwest Denmark, Esbjerg, Denmark; 4 Department of Medicine, Doherty Institute, University of Melbourne, Melbourne, Australia; 5 Simpson Centre for Reproductive Health, Edinburgh Royal Infirmary, Edinburgh, United Kingdom; 6 Department of Maternity, National Hospital Simão Mendes, Bissau, Guinea-Bissau; 7 Department of Public Health Sciences, Division of Global Health (IHCAR), Karolinska Institute, Stockholm, Sweden; 8 Institute of Clinical Research, OPEN, University of Southern Denmark / Odense University Hospital, Odense, Denmark; Univesity of Iowa, UNITED STATES

## Abstract

**Background:**

Stillbirth rates remain high in many low-income settings, with fresh (intrapartum) stillbirths accounting for a large part due to limited obstetrical care. We aimed to determine the stillbirth rate and identify potentially modifiable factors associated with stillbirth in urban Guinea-Bissau.

**Methods:**

The study was carried out by the Bandim Health Project (BHP), a Health and Demographic Surveillance System site in the capital Bissau. We assessed stillbirth rates in a hospital cohort consisting of all deliveries at the maternity ward at the National Hospital Simão Mendes (HNSM), and in a community cohort, which only included women from the BHP area. Stillbirth was classified as fresh (FSB) if fetal movements were reported on the day of delivery.

**Results:**

From October 1 2007 to April 15 2013, a total of 38164 deliveries were registered at HNSM, among them 3762 stillbirths (99/1000 births). Excluding deliveries referred to the hospital from outside the capital (9.6%), the HNSM stillbirth rate was 2786/34490 births (81/1000). During the same period, 15462 deliveries were recorded in the community cohort. Of these, 768 were stillbirths (50/1000).

Of 11769 hospital deliveries among women from Bissau with data on fetal movement, 866 (74/1000) were stillbirths, and 609 (70.3%) of these were FSB, i.e. potentially preventable. The hospital FSB rate was highest in the evening from 4 pm to midnight (P = 0.04). In the community cohort, antenatal care (ANC) attendance correlated strongly with stillbirth reduction; the stillbirth rate was 71/1000 if the mother attended no ANC consultations vs. 36/1000 if she attended ≥7 consultations (P<0.001).

**Conclusion:**

In Bissau, the stillbirth rate is alarmingly high. The majority of stillbirths are preventable FSB. Improving obstetrical training, labour management (including sufficient intrapartum monitoring and timely intervention) and hospital infrastructure is urgently required. This should be combined with proper community strategies and additional focus on antenatal care.

## Introduction

The global burden of stillbirths remains unacceptably high, with 2.6 to 2.7 million stillbirths per year[1, 2], the vast majority occurring in low- and middle-income countries[3]. The highest rates are observed in Sub-Saharan Africa, with an average rate of 32/1000 deliveries[4], compared to e.g. 5/1000 deliveries in the United Kingdom[5].

Until recently stillbirths have received limited attention globally and were not part of the Millennium Development Goals[6]. Stillbirths are often not included in national or international birth statistics and therefore remain a largely hidden problem[6]. This lack of data hampers efforts to reduce stillbirths[7], even though a large part of stillbirths, especially intrapartum, are preventable through simple and affordable interventions[8].

We assessed the stillbirth rate in urban Guinea-Bissau over a six-year period (2007–2013). Very limited stillbirth data are currently available from Guinea-Bissau. A rural study focusing on maternal mortality found a stillbirth rate of 44/1000[9], while the World Health Organization (WHO) estimates the country’s overall stillbirth rate to be 30/1000[10].

The aim of the present study was to determine the stillbirth rate in both a hospital cohort and a community cohort. Furthermore, we aimed to compare the stillbirth rate among women residing in the Bandim Health Project (BHP) area (community cohort), where health surveillance is conducted on a frequent basis, versus women living in other parts of the capital Bissau. Finally, in the hospital cohort, using data on fetal movements on the day of delivery, we assessed the rate of, and possible reasons (risk factors) for, fresh stillbirths (FSB). i.e. potentially preventable intrapartum fetal deaths.

## Methods

### Setting

The study was conducted by the Bandim Health Project (BHP) in Guinea-Bissau (www.bandim.org). The BHP is a Health and Demographic Surveillance System (HDSS) site established in 1978 and now covering six suburbs and approximately 103,000 inhabitants in the capital Bissau. Over the years, the HDSS has provided data for hundreds of studies with a focus on childhood health, vaccines, infections, nutrition and predictors of child mortality. The BHP has a strong link to the Ministry of Health in Guinea-Bissau, ensuring that important findings can be readily communicated to the local health authorities.

The routine HDSS data collection consists of the following elements:

#### HDSS study area data collection

All women of childbearing age are visited monthly to identify new pregnancies and births. All children are followed up to until the age of three years. Censuses of the whole study population are carried out at 2–3 years intervals.

Within the study area, there are three health centres, providing basic antenatal care, child consultations and routine vaccinations. The BHP registers these consultations and pregnant women are regularly encouraged to attend antenatal care.

#### Hospital data collection

In the centre of Bissau, the BHP collects data at the maternity ward at the National Hospital Simão Mendes (HNSM), the country’s principal public referral hospital which is situated two kilometres outside the BHP study area (**S1 Fig)**. Many women from the BHP area give birth at the HNSM hospital[11].

According to the United Nations, Guinea-Bissau ranks among the poorest in the world[12].

### Hospital cohort

This cohort included births registered at the HNSM from October 1 2007 to April 15 2013, i.e. 5 years and 6½ months of data. Mothers are charged for delivery, including for caesarean section and medicines if needed. The maternity ward’s facilities are very modest, with frequent cuts of electricity and water supply.

Hospital data were collected by two methods: The hospital staff’s own registration and that of the BHP assistants working at the hospital.

#### Hospital’s own data registration

The hospital has its own register of deliveries. It includes date and time of birth; mode of delivery (vaginal vs. caesarean section); presentation (vertex or breech); weight; sex; number of fetuses; Apgar score; vital status of mother and newborn(s); maternal age and ethnicity; residential area. A partogram is usually also completed.

From February 2011, the timing of last felt fetal movements was registered for a subset of women. Fetal heart rate (FHR) was recorded for a subset, but many records had missing information and we could not ascertain whether FHR was zero or merely not measured.

#### BHP data registration at the hospital

Every morning, a BHP research assistant collected additional information of all hospital births, including maternal vital status, civil status, educational level and current and previous pregnancies.

In 2012, questions regarding antenatal ultrasound scans (usually carried out at private clinics) were added. The assistant confirmed whether any infants recorded as stillbirth by the delivery room staff had in fact died.

#### HIV testing at the hospital

Since July 2008, all women giving birth at the HNSM were offered free HIV testing, though test kit stock-outs sometimes occurred. A rapid diagnostic test Determine^®^ (Abbot Diagnostics, Maidenhead, United Kingdom) was used for screening, and positive results were confirmed by SD Bioline HIV-1/2 (Standard Diagnostics, Kyonggi-do, South Korea). Antiretroviral therapy was administered to prevent vertical transmission.

#### Hospital deliveries among women from outside Bissau

Approximately one tenth of the women giving birth at the HNSM were registered as residing outside Bissau. We had limited data on the decision process regarding travelling to Bissau to give birth. Hence, we considered these women as “referred”.

### Community cohort

Hospital data can be biased due to selected referrals of high-risk pregnancies and the inclusion of more women of higher socio-economic status[13], and it is therefore important to also examine stillbirths in a community context[14]. Thus, the BHP registered deliveries and stillbirths occurring in its study area. Drawing on HDSS data, we defined a “community cohort” covering the same period as the “hospital cohort”, i.e. from October 1 2007 to April 15 2013. Since there is a risk of postpartum registration of deliveries capturing live births selectively, we only included women in the community cohort who had been registered as pregnant prior to delivery.

Many of the HDSS information variables were the same as at the hospital; additional information was available regarding socio-economic characteristics and antenatal care (ANC), whereas time of birth, Apgar score, partogram data and HIV status were not available for the community cohort.

#### Overlap with hospital cohort

**Fig 1:**
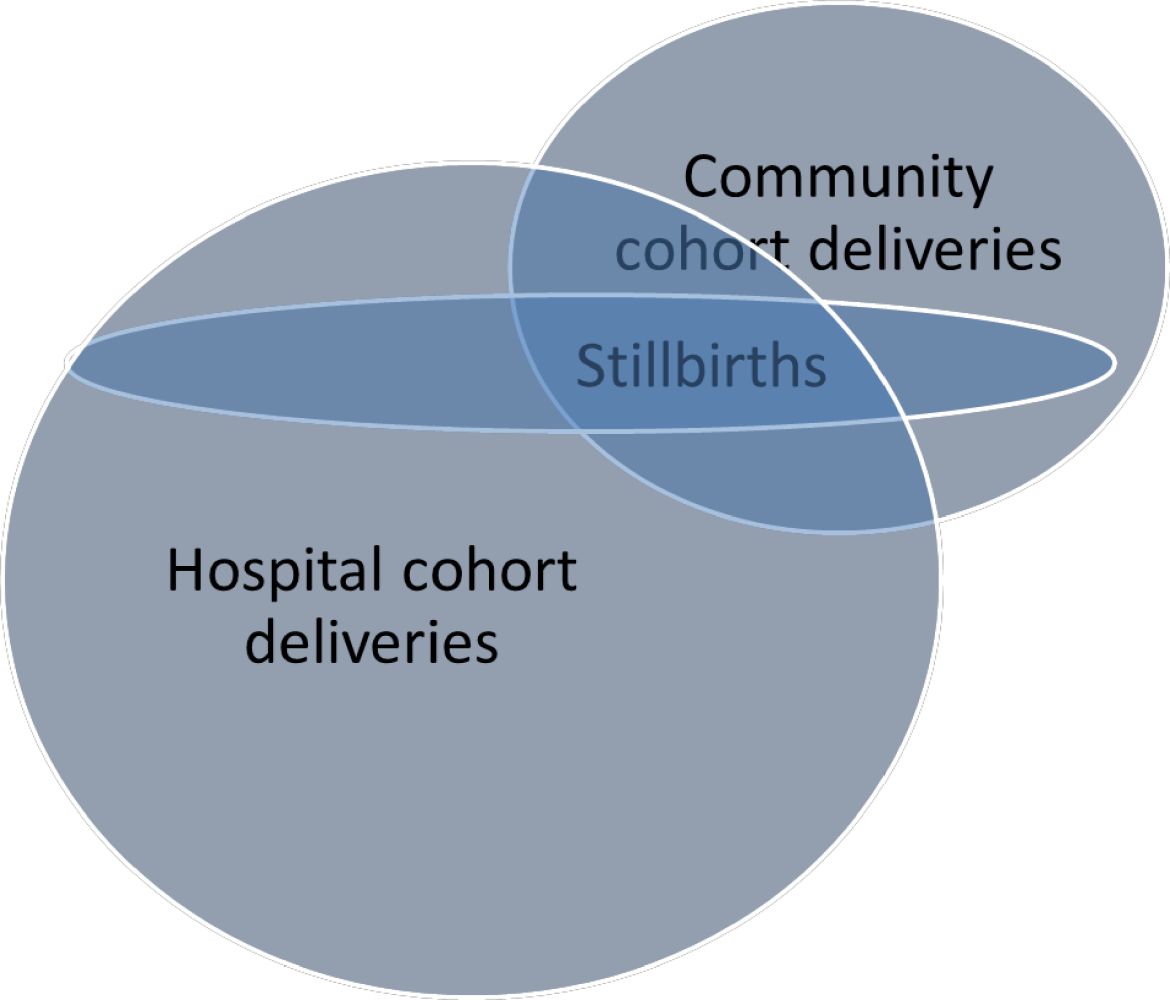
Overlap between births from the BHP study area (community cohort) and births at the HNSM hospital. The figure illustrates that there was a substantial overlap between the two cohorts, i.e. a significant proportion of community cohort births occurred at the HNSM.

A substantial proportion of the mothers from the community cohort gave birth at the HNSM, resulting in an overlap (see **Fig 1**). These deliveries were thus included in both the community cohort and hospital cohort stillbirth rates. For 0.48% (29/5995) of children in the community cohort that ultimately delivered at HNSM there were discrepancies between hospital and community stillbirth records. Here, the hospital recordings were used.

### Stillbirth definitions

The stillbirth burden was defined as stillbirths per thousand total births (denominator). Miscarriages (birth weight <1000 g) were excluded. We use the word “births” synonymously with “number of fetuses at risk”. Hence, the total number of pregnant women was slightly lower than the total number of births, due to twin and triplet gestations.

Stillbirth was classified as FSB if fetal movements were reported on the day of delivery. FSB is opposed to antepartum fetal death, often referred to as macerated stillbirth (MSB) due to its dermatological appearance, which indicates death for at least 12–24 hours [15]. We focused particularly on FSB as it is often caused by intrapartum asphyxia due to e.g. prolonged labour, lack of fetal monitoring and delays in timely delivery, and can thus be prevented through appropriate management of labour and delivery. Antepartum deaths, which are more difficult to prevent, can be due to many fetal and maternal causes, including congenital anomalies, infections, diabetes, hypertension, placental abruption or insufficiency and intrauterine growth restriction [6].

Due to different limitations in the data collection methods for the two cohorts it was necessary to define stillbirth slightly differently in each cohort:

#### Hospital cohort

At the HNSM, we were able to define stillbirth in accordance with the WHO criteria as data on vital status and birthweight were routinely recorded, i.e. a newborn at or above 1000 g showing no vital signs immediately after birth (Apgar score = 0)[6]. A total of 32 newborns had been recorded as stillborn by the hospital staff, even though a positive Apgar score was registered. These infants (0.85% of total stillbirths) were re-classified as live born.

#### Community cohort

We defined stillbirth as an infant showing no signs of life at birth, as birthweight and fetal movements were not routinely recorded. We were unable to assess factors associated with FSB in women delivering outside the HNSM.

### Statistical methods

Statistical analyses were carried out using STATA software v.11 (Stata Corporation, College Station, TX, USA). Trend over time was determined using logistic regression, with year of birth as a continuous variable.

We investigated possible risk factors associated with FSB (i.e. presumably preventable stillbirths) among hospital deliveries from Bissau, based on previously reported risk factors [3, 4, 7, 13–19]. The following were included: maternal age, ethnicity, civil status, educational level, sex, twin gestation, birth weight (<2500g, 2500–4000g, >4000g), breech presentation, previous stillbirth and time of delivery during the day (8 a.m.-4 p.m., 4 p.m.-midnight and midnight-8 a.m.). Children born by caesarean section were not excluded from the statistics, but we did not include caesarean section as a variable in our main risk factor model, as the procedure is an indicator of obstetrical problems, potentially leading to FSB. Neither did we include parity due to collinearity with maternal age. We did, however, conduct a secondary risk factor analysis which included the variable “Caesarean section”, in order to determine the strength of the association between the procedure and FSB.

Risk factor analyses were done using logistic regression, providing odds ratios (OR). We conducted both a univariate and a multivariate analysis. Adjustments were made for the clustering of twins, using a *pair* number. Missing values were rare and hence no “missing” categories were introduced in the statistical model for each variable.

Characteristics for hospital vs. non-hospital births and hospital births from the BHP area vs. rest of Bissau were compared using Poisson regression with robust variance estimation providing prevalence ratios (PR) for categorical variables. Continuous variables were compared using Student’s t-test if normally distributed or Mann-Whitney test if non-normally distributed.

Unless otherwise specified, we excluded women referred from outside the capital Bissau, as their referral very likely was due to obstetrical problems.

### Ethical considerations

The BHP collects data at the National Hospital and in the BHP study area at the request of the Guinean Ministry of Health, with no additional (written) consent required. Participating women gave oral consent to the collection of basic pregnancy and birth related data. The study protocol was approved by the National Health Ethical Committee (Comité Nacional de Ética na Saúde) in Guinea-Bissau (CNES-2010-018). Separate ethical approval from the same institution was obtained for the linkage with maternity HIV data (CNES-2011-030).

## Results

### Characteristics of the women giving birth

#### Hospital vs. non-hospital deliveries

Amongst women in the community cohort, 5995 births (38.8%) occurred at the HNSM whereas the remaining 9467 infants were born elsewhere, i.e. either at home or at small birth facilities. The proportion of women in the community cohort that delivered at HNSM declined during the study period (P<0.001). Mothers giving birth outside the hospital were more often of Pepel ethnicity, had higher parity, had fewer ANC visits and had poorer socio-economic indicators (P<0.001). Twinning was more common at the hospital (PR = 1.46, 95% CI: 1.22–1.74) (see Table 1).

**Table 1:** Characteristics among HNSM vs. Non-HNSM deliveries from the community cohort.

	At HNSM N = 5995	Outside HNSM N = 9467	P or Prevalence Ratio (95% CI)
**Maternal**			
Age; years [N = 14916]	25.1 (5.81)	25.0 (5.75)	0.69
Ethnicity; %			
Balanta	8.2% (492/5990)	8.1% (762/9439)	1.02 (0.91–1.14)
Fula	23.2% (1390/5990)	26.2% (2471/9439)	0.89 (0.83–0.95)
Pepel	21.3% (1278/5990)	30.6% (2890/9439)	0.70 (0.65–0.75)
Mandinga	8.7% (521/5990)	7.2% (678/9439)	1.21 (1.08–1.36)
Manjaco	11.7% (703/5990)	9.9% (935/9439)	1.19 (1.08–1.31)
Mancanha	9.1% (546/5990)	4.6% (433/9439)	1.99 (1.76–2.26)
Other	17.7% (1060/5990)	13.5% (1270/9439)	1.32 (1.21–1.43)
Parity [N = 15457]*	2 (1–3)	2 (1–4)	<0.001
Last born child still alive; %	93.5% (3226/3452)	95.4% (5693/5968)	0.98 (0.94–1.02)
No. of ANC visits [N = 15251]*	4 (2–6)	4 (1–5)	<0.001
Education; %			
No schooling	21.1% (1186/5614)	31.4% (2566/8169)	0.67 (0.63–0.72)
1–4 years	9.8% (549/5614)	15.5% (1264/8169)	0.63 (0.57–0.70)
≥5 years	69.1% (3879/5614)	53.1% (4339/8169)	1.30 (1.25–1.36)
Married; %	64.1% (3841/5998)	67.4% (6350/9423)	0.95 (0.91–0.99)
House has electricity; %	40.3% (2413/5986)	20.6% (1947/9451)	1.96 (1.84–2.08)
House has television; %	50.6% (3027/5980)	21.3% (2012/9447)	2.38 (2.25–2.51)
**Infant**			
Male sex; %	53.0% (3173/5991)	51.2% (4839/9450)	1.03 (0.99–1.08)
Twin; %	3.9% (231/5995)	2.6% (250/9467)	1.46 (1.22–1.74)
Birth weight; g [N = 11897]	3088 (560)	3100 (550)	0.26

Cells are percent (n/N) or mean (SD), except for continuous non-normally distributed data (marked with *) which are presented as median (25%-75% percentiles). For continuous data, we have added the total number [N] of observations.

#### Hospital births among women from the BHP area (community cohort) vs. the rest of Bissau

Women from other parts of the capital Bissau were younger (P<0.001), more often of Balanta ethnicity (PR = 2.78, 95% CI: 2.54–3.00), more likely to be married (1.04, 1.00–1.07), HIV-infected (1.32, 1.12–1.55) and without formal schooling (1.15, 1.08–1.22), when compared with women from the community cohort (BHP) area (**S1 Table**).

### Stillbirth rates

#### Overall stillbirth rates

**Fig 2:**
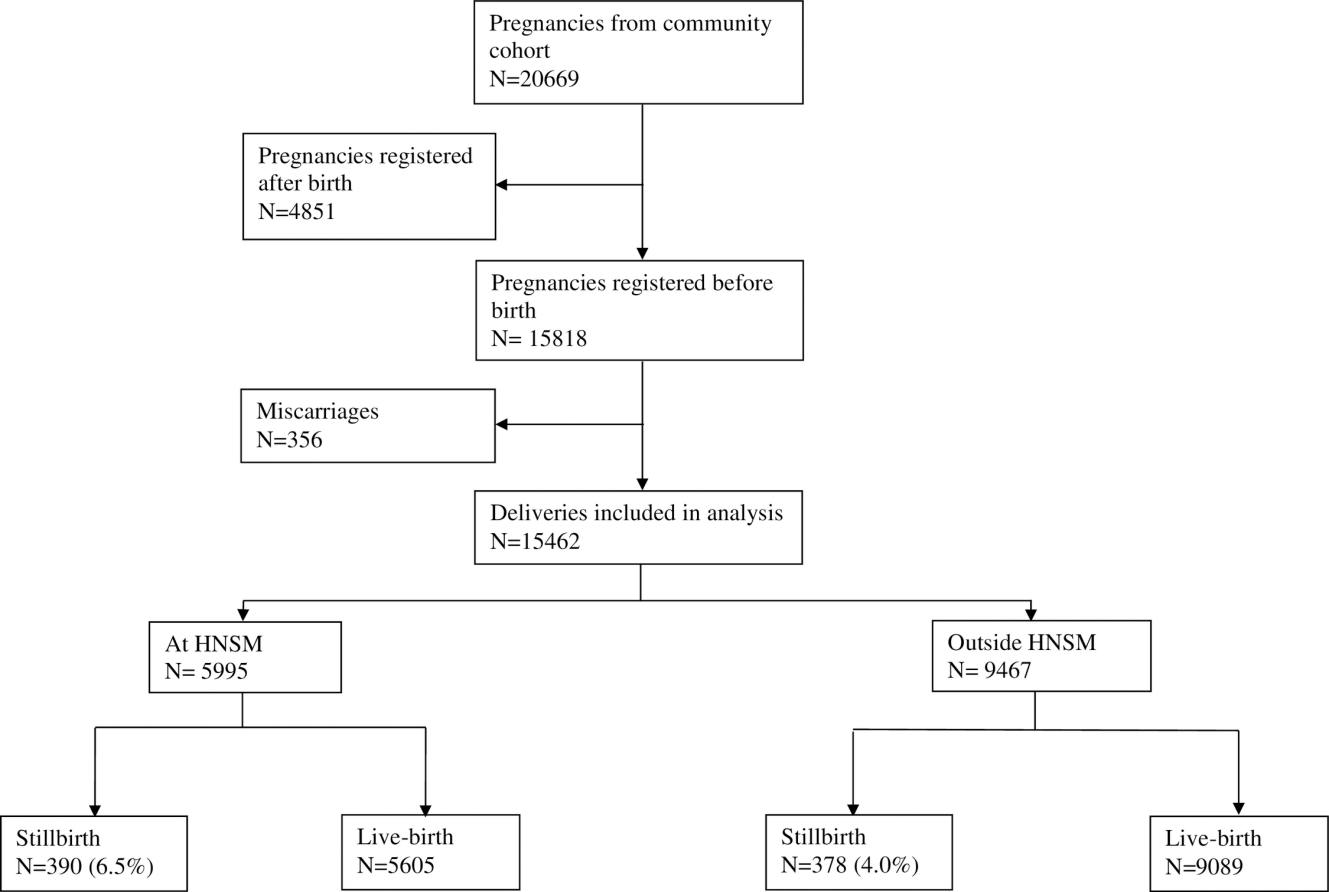
Community cohort (BHP area) deliveries. The figure illustrates births and stillbirths in the community cohort, including how many occurred at the HNSM and outside the hospital.

From 2007 to 2013 there were 15462 births in the community cohort, including 768 stillbirths (see **Fig 2**). The overall community stillbirth rate was 50/1000 (768/15462), with a tendency to decline during the study period (P = 0.07) (Table 2). During the same period, we registered 38164 births at the HNSM hospital, with 6723 of these (17.6%) being from the community cohort area. There were 3762 stillbirths at HNSM, resulting in an overall hospital stillbirth rate of 99/1000 (3762/38164). The rate did not change significantly over time (P = 0.93).

**Table 2:** Stillbirth and caesarean section rates in the community and hospital cohorts.

	2007	2008	2009	2010	2011	2012	2013	Total	P for trend
**Community cohort**									
Stillbirth rate	51 (44/857)	55 (167/3057)	50 (138/2774)	51 (135/2683)	51 (144/2834)	43 (116/2678)	42 (24/579)	50 (768/15462)	0.07
C-section rate	53 (45/853)	53 (161/3048)	49 (136/2765)	55 (146/2668)	68 (193/2826)	60 (159/2661)	91 (52/569)	58 (892/15390)	<0.001
Birth at hospital	49.7% (426/857)	42.4% (1295/3057)	39.4% (1094/2774)	42.0% (1128/2683)	35.5% (1001/2834)	32.5% (870/2678)	31.3% (181/579)	38.8% (5995/15462)	<0.001
**Hospital cohort**									
Stillbirth rate overall	91 (206/2254)	98 (741/7559)	103 (728/7082)	100 (714/7064)	97 (638/6597)	95 (569/5997)	103 (166/1611)	99 (3762/38164)	0.93
Stillbirth rate, community cohort area	57 (27/472)	61 (91/1485)	55 (67/1230)	60 (76/1274)	60 (66/1102)	53 (51/964)	46 (9/196)	58 (387/6723)	0.52
Stillbirth rate, other parts of Bissau	76 (118/1544)	88 (469/5355)	87 (443/5107)	92 (475/5182)	85 (419/4926)	83 (364/4412)	89 (111/1241)	86 (2399/27767)	0.98
Stillbirth rate among mothers from outside Bissau	256 (61/238)	252 (181/719)	293 (218/745)	268 (163/608)	269 (153/569)	248 (154/621)	264 (46/174)	266 (976/3674)	0.75
C-section rate	120 (270/2251)	134 (1009/7554)	120 (848/7043)	139 (983/7064)	167 (1103/6597)	188 (1130/5997)	178 (287/1611)	148 (5630/38117)	<0.001
From community cohort	20.9% (472/2254)	19.7% (1485/7559)	17.4% (1230/7082)	18.0% (1274/7064)	16.7% (1102/6597)	16.1% (964/5997)	12.2% (196/1611)	17.6% (6723/38164)	<0.001

Stillbirth and caesarean section rates are displayed per 1000 births (n/N). The percentage of community cohort births at the hospital are displayed as percent (n/N). Note: In the hospital cohort, the deliveries from the community cohort area included pregnancies only registered after delivery; thus, the hospital cohort’s N for deliveries “From community cohort” are slightly higher than the N for the community cohort’s “Birth at hospital”.

Combing the two cohorts (community cohort deliveries both at the HNSM and outside and hospital deliveries from the rest of the capital Bissau), the stillbirth rate was 73/1000 (3167/43229).

#### Hospital stillbirths among women in the BHP area vs. rest of the capital Bissau

**Fig 3:**
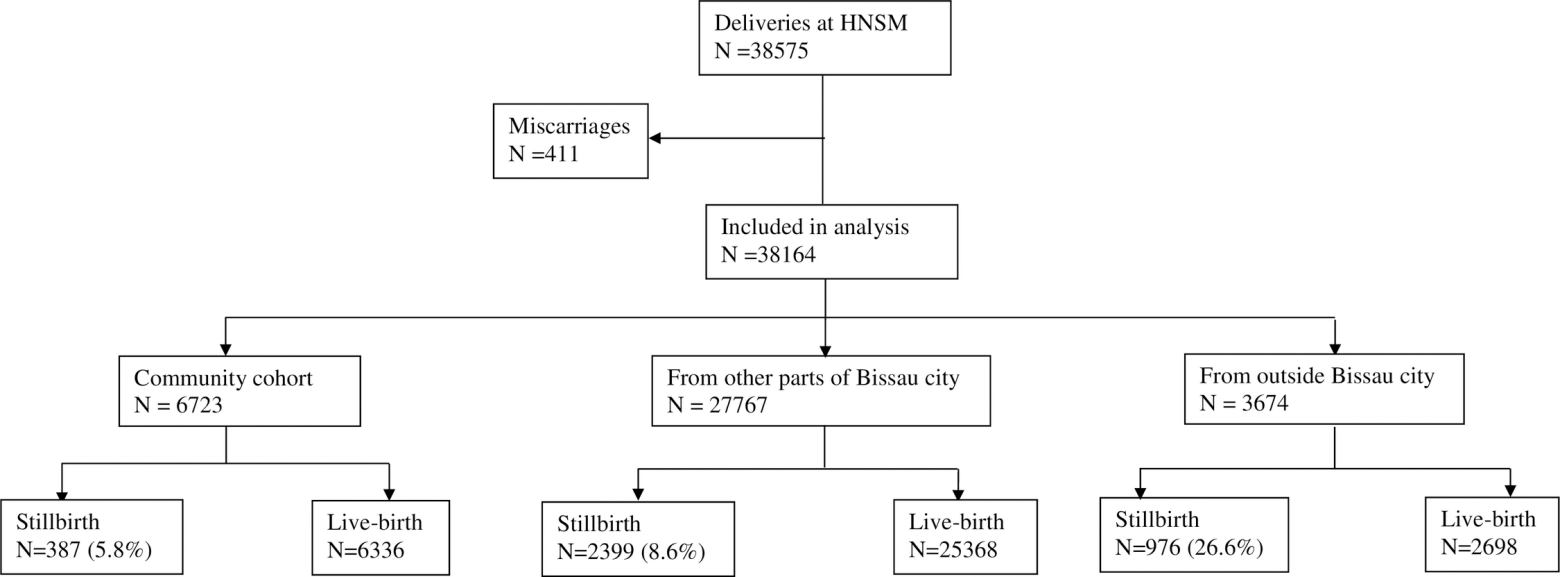
HNSM hospital cohort births from the community cohort (BHP area), rest of Bissau and outside Bissau. The figure illustrates births and stillbirths at the HNSM hospital, including how many were from the community cohort, the rest of the capital Bissau and outside Bissau.

The hospital stillbirth rate was 58/1000 (387/6723) among mothers residing in the BHP area while it was 86/1000 (2399/27767) among women from the rest of the capital Bissau (combined Bissau stillbirth rate 81/1000 [2786/34490]) (see **Fig 3**). No time trends were observed.

#### Hospital stillbirths among women referred from outside Bissau (rural areas)

Among women referred to HNSM from outside of Bissau (9.6% of total hospital births), the overall hospital stillbirth rate was 266/1000 (976/3674). The hospital stillbirth rate was even higher amongst women travelling to HNSM from the most remote parts of Guinea-Bissau, e.g. 373/1000 (84/225) for women from the Gabu region.

#### Antenatal care and stillbirths in the community cohort

In the community cohort, data on antenatal care (ANC) attendance was available. The number of ANC visits was inversely correlated to stillbirths (P for trend<0.001) (**S2 Fig**). The stillbirth rate was 71/1000 if the mother never attended ANC vs. 36/1000 if she had attended seven times or more.

#### HIV status and hospital stillbirths

Five percent (1174/22109) of the women from Bissau being tested were HIV-infected. A total of 115 women declined HIV testing. The overall stillbirth rate was 82/1000 (1707/20935) for HIV-uninfected mothers and 95/1000 (112/1174) for HIV-infected (unadjusted OR = 1.19 [0.97–1.46]).

#### Ultrasound scan and hospital stillbirths

Information on antenatal ultrasound scan was available for 5372 hospital deliveries from Bissau (2012–2013). The unadjusted stillbirth rate was lower if the mother had been scanned (0.47, 0.37–0.60). Adjusted for ethnicity, education and marital status the association was weaker (0.62, 0.47–0.81).

### FSB and its risk factors (hospital cohort)

#### FSB rate

In the hospital cohort, and amongst women who resided in Bissau, data on fetal movement were available for 11769 deliveries. Among these, there were 866 stillbirths, of which 609 were classified as FSB (70.3%), translating into a FSB rate of 52/1000.

#### Main risk factor analysis

Our final adjusted multivariate model included 9854 births. Compared with being 21–25 years old, the odds of FSB increased with advancing maternal age, i.e. between 31–35 years the OR was 1.70 (95% CI = 1.22–2.39) (Table 3).

**Table 3:** Risk factors of fresh stillbirth (FSB) at the HNSM hospital. Women from outside Bissau city excluded.

		FSB rate/1000 (n/N)	Univariate OR (CI)	Multivariate OR (CI) N = 9854
**Overall**				
**Maternal factors**				
Age	≤ 16 years	63 (32/511)	1.39 (0.94–2.06)	1.24 (0.76–2.02)
	17–20 years	52 (147/2838)	1.14 (0.90–1.44)	1.16 (0.86–1.54)
	21–25 years	46 (146/3186)	1.00 (ref)	1.00 (ref)
	26–30 years	51 (151/2944)	1.13 (0.89–1.42)	1.17 (0.88–1.55)
	31–35 years	66 (87/1324)	1.46 (1.11–1.93)	1.70 (1.22–2.39)
	36–40 years	71 (37/521)	1.59 (1.10–2.31)	1.51 (0.96–2.38)
	> = 41 years	32 (1/31)	0.69 (0.09–5.13)	1.25 (0.16–9.71)
Ethnicity	Balanta	65 (161/2497)	1.00 (ref)	1.00 (ref)
	Fula	63 (157/2489)	0.98 (0.78–1.23)	0.80 (0.60–1.08)
	Pepel	40 (61/1526)	0.60 (0.45–0.82)	0.64 (0.46–0.89)
	Mandinga	51 (63/1247)	0.77 (0.57–1.04)	0.66 (0.46–0.95)
	Manjaco	41 (37/893)	0.63 (0.43–0.91)	0.68 (0.45–1.00)
	Mancanha	35 (33/941)	0.53 (0.36–0.77)	0.56 (0.37–0.85)
	Other	54 (97/1806)	0.82 (0.63–1.07)	0.77 (0.57–1.05)
Civil status	Single	50 (162/3238)	1.00 (ref)	1.00 (ref)
	Married	53 (428/8120)	1.06 (0.88–1.27)	0.75 (0.58–0.97)
Educational level	No schooling	84 (129/1531)	1.00 (ref)	1.00 (ref)
	1–4 years	50 (81/1611)	0.58 (0.43–0.77)	0.56 (0.41–0.77)
	≥5 years	39 (268/6855)	0.44 (0.36–0.55)	0.45 (0.35–0.59)
**Infant factors**				
Sex	Male	58 (345/6002)	1.00 (ref)	1.00 (ref)
	Female	49 (263/5391)	0.84 (0.71–0.99)	0.80 (0.66–0.97)
Twin	No	54 (583/10871)	1.00 (ref)	1.00 (ref)
	Yes	49 (26/527)	0.92 (0.59–1.42)	0.48 (0.29–0.82)
Birth weight	<2500 g	151 (244/1614)	4.67 (3.92–5.57)	4.57 (3.68–5.68)
	2500–4000 g	37 (344/9371)	1.00 (ref)	1.00 (ref)
	>4000 g	52 (21/406)	1.43 (0.91–2.25)	1.59 (0.99–2.57)
**Obstetric factors**				
Fetal presentation	Vertex	53 (599/11318)	1.00 (ref)	1.00 (ref)
	Breech	78 (5/64)	1.52 (0.60–3.83)	0.37 (0.04–3.11)
Previous stillbirth	No	50 (341/6777)	1.00 (ref)	1.00 (ref)
	Yes	102 (29/284)	2.17 (1.45–3.24)	1.83 (1.17–2.88)
	No previous birth	49 (204/4185)	0.97 (0.81–1.16)	1.00 (0.76–1.31)
**External factors**				
Time of delivery*	8 a.m.-4 p.m.	55 (196/3578)	1.00 (ref)	1.00 (ref)
	4 p.m.-midnight	64 (220/3414)	1.19 (0.97–1.45)	1.27 (1.00–1.61)
	Midnight-8 a.m.	44 (192/4353)	0.80 (0.65–0.98)	0.87 (0.68–1.10)

*24-hour clock

Women from the Balanta ethnic group had a higher FSB rate compared to women from all other ethnic groups (OR 1.42; 1.14–1.76). A history of stillbirth was also a FSB risk factor (1.83, 1.17–2.88).

For birth weight below 2500 g the risk of FSB was considerably higher (4.57, 3.68–5.68), while a tendency towards increased risk was observed for birth weight above 4000 g (1.59, 0.99–2.57).

Married women had a lower FSB rate (0.75, 0.58–0.97). Any maternal education, 1–4 years (0.56, 0.41–0.77) and ≥5 years (0.45, 0.35–0.59), was a highly protective factor.

The adjusted FSB rate was lower for female fetuses (0.80, 0.66–0.97) and for twins (0.48, 0.29–0.82).

Time of delivery during the day between 4 p.m. to midnight, compared with 8 a.m. to 4 p.m., was a risk factor (1.27, 1.00–1.61). The proportion of FSB was highest around 8 p.m. (82/1000) and lowest around 2 a.m. (30/1000) (**S3 Fig**). Most births occurred at night (Table 3).

#### Secondary risk factor analysis including caesarean section

In a separate model including caesarean section as a risk factor variable, the procedure was strongly correlated with FSB (2.46, 2.05–2.96). The proportion of caesarean sections increased at the hospital during the study (P<0.001).

## Discussion

### Main findings

In Bissau, Guinea-Bissau, we observed very high stillbirth rates, both in the hospital and community setting. At the National Hospital, the stillbirth rate was 99/1000 overall and 81/1000 for women residing in the capital Bissau. The community stillbirth rate of 50/1000 also exceeded what has been found in most of Africa[7]. FSBs, i.e. potentially preventable intrapartum fetal deaths, accounted for 70% of all hospital stillbirths. Lack of antenatal care, poor maternal education and possibly also sub-standard hospital delivery routines were identified as potentially modifiable factors in stillbirth prevention.

### Strengths and limitations

The main strengths of this study were the prospective data collection and the large sample size, both at community and hospital levels. Amongst others, this also enabled us to examine time trends.

The study depends on a correct classification of stillbirths. Misclassification could be a problem, i.e. early neonatal deaths may have been recorded as stillbirths[20]. Reasons for misclassification by health personnel include lack of knowledge, inadequate assessment of vital signs and fear of blame[15]. The measurement of Apgar score also has some uncertainty, as it is often done by mere clinical impression, not strict criteria. Approximately one percent of the children registered as stillborn by the hospital staff actually had a positive Apgar score. In our hospital analyses, we deliberately chose a strict stillbirth definition, which included children with a positive Apgar score as livebirths. Importantly, there was almost no discrepancy between hospital and community data with regard to stillbirth, making it unlikely that there was a high degree of misclassification.

In the community cohort, birth weight was not available for all women and some miscarriages may have been categorised as stillbirths; community stillbirth rates may have been overestimated as a result.

We had limited data on the referral or decision process concerning hospital births. Some women may have planned to give birth at home or a local health center, but then had been referred to the HNSM due to labour problems. Others may have planned to give birth at the hospital all along.

FSB was defined by maternal reporting of fetal movements. Reduced fetal movements is correlated with stillbirth[21, 22]. FHR was not used, as we could not distinguish between missing values and cases where FHR was truly zero. The stillbirth rate was 69.5% if fetal movements had not been felt by the mother for at least one day prior to delivery (data not shown), providing some validation of our method. Dermatological assessment of the skin in order to classify stillbirths was not available but was recently found to be inaccurate in a study from Ghana[23].

Fetal movements were only recorded from 2011 to 2013. If emergency obstetric care had improved at the hospital over the years, this could mean that our FSB:MSB ratio would not be representative for the entire study period. Likewise, HIV status was not available for the entire hospital cohort due to e.g. periods of stock-outs of test kits. However, by our impression, the hospital conditions did not change much over the years, nor was any trend observed in the hospital stillbirth rate. Hence, we consider our results representative for the study period.

As we did not have unique maternal ID numbers for the entire hospital dataset and the mothers’ names were often spelled slightly different on various occasions, we could not adjust for mothers giving birth more than once during the study. Among women in the community cohort (all registered with unique BHP IDs), 8.5% gave birth twice during the study, while 0.5% gave birth three times.

Finally, data were not available on maternal lifestyle and sexually transmitted diseases other than HIV, though such factors can affect stillbirth rates.

### Interpretation

The hospital stillbirth rate among women from Bissau was very high, likely reflecting problems with early identification of high-risk pregnancies, timely referral and also sub-optimal care during labour and delivery.

At the HNSM, 70% of the stillbirths with fetal movement data were FSB, i.e. deaths potentially preventable by more comprehensive midwifery and obstetric care. Our figures correspond to a FSB:MSB ratio of 2.4:1. A hospital study from neighbouring The Gambia observed a FSB:MSB ratio of 1.3:1[17] while in rural Central Africa it was 4:1[15]. In high-income countries, the vast majority of stillbirths occur antepartum[24], reflecting better antenatal and comprehensive emergency obstetric care.

In other low-resource African settings such as Malawi and Zambia, community stillbirth rates of 40-50/1000 have been reported[13]. A population-based multicentre study from six West African urban sites reported an average stillbirth rate of 26/1000[25]. Thus, our community stillbirth rate was very high.

The fact that the hospital stillbirth rate was even higher than the community rate was likely due to the HNSM being the major referral unit for complicated pregnancies and births. Reasons for transferral often included obstructed labour and severe haemorrhage.

Though difficult to ascertain, sub-standard clinical routines and human factors could also play a role. Time of delivery during the day was associated with altered FSB risk: rates peaked around 8 pm, and to a lesser extent around 8 am and 3 pm. Since most hospital births occurred later at night, it is unlikely that the midwives and nurses would be most busy around 8 pm, but shift handovers or meal times may have played a role. Though the time of fetal death was unknown, it is noteworthy that the lowest rate was observed at 2 a.m., when the midwives would presumably be more tired.

At the adjacent paediatric ward, the mere presence of dedicated staff has been associated with lower child mortality[26, 27].

Caesarean sections increased at the hospital over time, possibly reflecting changes in policy and staff or more complicated births. The WHO estimates that a community caesarean section rate of 100/1000 is necessary to reduce perinatal mortality[28]. Thus, our community rate of 58/1000 may be too low, though the main objective is providing caesarean sections to those in need[28]. In a very resource-limited setting such as Bissau, caesarean section is connected with a number of risks both in the acute phase (anaesthetic complications, bleeding, infection, delays with transfer to theatre) and in the longer term (uterine scar dehiscence and its detrimental sequelae in a future pregnancy). Thus, while prompt caesarean section can be life-saving, improvements in labour management (e.g. correct partograph use), limiting second stage labour and the judicious and competent use of instrumental delivery in lieu of caesarean section may be equally important. Of some concern, the increase in hospital sections did not coincide with fewer hospital stillbirths. As the community caesarean section rate was largely driven by sections carried out at the HNSM (the largest obstetrical referral centre in Guinea-Bissau), we cannot exclude that in some cases caesarean section was performed at the HNSM without clear clinical indication, while at the same time being underused at other minor health facilities outside the HNSM.

Concerning factors associated with FSBs, which can be considered potentially preventable fetal deaths, previously reported maternal risk factors for FSB such as advanced age[4], low education[19] and single marital status[19] were confirmed. Interestingly, the FSB rate was higher for the Balanta ethnic group, which may reflect cultural, geographical or genetic factors.

Low birth weight (<2500g) increased the odds of FSB fourfold. These fetuses probably have limited reserves to withstand birth asphyxia due to pre-existing growth restriction and/or prematurity. For large fetuses (>4000g) the mechanism is likely to be labour obstruction. In line with a recent large-scale British study[29], female fetuses were at lower FSB risk. Previous stillbirth was also strongly associated with FSB[30]. Causes of recurrent stillbirth include congenital and genetic disorders, placental and umbilical abnormalities, infections and hypertensive disease of pregnancy [31].

In the adjusted model, twins had a significantly lower FSB rate than singletons. This was surprising, as one would expect more complications related to multiple gestations[32], but to some extent it relates to the adjustment of confounders such as low birth weight and breech presentation. Other researchers from Sub-Saharan Africa found no significant excess stillbirth rate for twins[15]. Twins have a high overall perinatal mortality in Bissau[11], which may be driven by early neonatal deaths rather than stillbirths. Furthermore, second trimester twin fetal demise due to e.g. twin-to-twin transfusion syndrome may not be captured using our birthweight cut-off to define miscarriage [33].

Finally, maternal HIV infection may increase the stillbirth rate due to opportunistic infections[34]; we also found that stillbirths tended to be more common in case of HIV-infection. HIV infection is highly stigmatized in Guinea-Bissau, which may also influence pregnancy outcomes.

In a separate multivariate model, caesarean section was strongly correlated with FSB. Caesarean section is a proxy for complicated births, and referral for caesarean section may often happen too late[35]. There may also be significant delays from time from the diagnosis of fetal distress to caesarean section.

An excessive use of oxytocin to expedite labour has been identified as a risk factor elsewhere[36]. We did not have data on this, but this may be of importance at HNSM, as remuneration of midwives was partly based on the number of births per shift.

### Perspectives for stillbirth prevention

An important aspect of the study was identifying potentially modifiable causes (risk factors) of stillbirth. We chose to focus on FSBs, as they are more often preventable. To some extent, stillbirth prevention can be divided into person-related factors, institutional issues and the factors related to the broader health care system available to pregnant women.

At a person-related level, FSBs were highly correlated to poor maternal education, indicating that this may well be crucial. Better educated women may be more likely to seek and regularly attend antenatal care and speak up if things do not go as planned. They may also be in a better position to raise finances for acute treatment. Finally, education is also important in terms of family planning. An equity based approach has previously been identified as key in stillbirth prevention[37].

At an institutional level, the high hospital stillbirth rate, including the very high rate of likely intrapartum deaths (FSBs), indicates that significant benefits could be obtained if hospital midwifery and comprehensive emergency obstetric care at referral centers such as the HNSM were improved [18, 38]. This should be combined with a thorough monitoring of procedures during labour and birth, and an evaluation of delays with receiving emergency care provision at the HNSM. This type of institutional delay has been referred to as Type 3-delay in the “three layer delay model”[39, 40]. In terms of training, programmes such as the “Helping Babies Breathe” initiative have demonstrated remarkable results in Tanzania[41]. Training should also focus on consistent use of partograms, frequent monitoring of fetal heart rate, as well as operative procedures such as vacuum extraction. Perinatal audit has also proved effective elsewhere[42]. Importantly, the fact that the mothers needed to pay for hospital services likely often caused fatal delays in acute situations. In other African settings, payment issues are only resolved after birth, which should be considered at the HNSM. Finally, it needs to be determined if cesarean sections are sometimes carried out at the HNSM without being strictly necessary.

When looking at the broader health care system, there are also several factors to address in stillbirth prevention. Importantly, the hospital stillbirth rate was lower among women from the community cohort, even after adjusting for various background factors. Here, the extensive community strategies provided by the BHP could have a positive impact on the stillbirth rate, by encouraging regular ANC attendance and improving health awareness. ANC represents a good opportunity to recognize high risk pregnancies[18, 38]. By raising birth preparedness, improved ANC also limits Type 1-delay stillbirths resulting from late decision to seek hospital medical care, thereby preventing FSBs. Thus, there was a clear inverse association between ANC visits and stillbirths. Proper ANC also includes malaria prevention, syphilis treatment and identification of hypertensive disorders of pregnancy and diabetes[38]. Particular attention should be devoted in case of previous stillbirth. Though technically more demanding, antenatal ultrasound scan could be of value[43]. Thus, the stillbirth rate was lower if the mother had been scanned, though some socio-economic confounding was present. Pre-labour caesarean sections for potentially complicated deliveries could be important[44], though the procedure should be used judiciously.

Transportation issues, including from rural areas, also need to be addressed (Type 2-delay). Distance to hospital, especially at night, has been recognised as a stillbirth risk factor[36, 45]. Thus, the stillbirth rate was more than 30% among transferrals from the most remote areas. This indicates that the obstetric services also need much improvement outside Bissau.

Finally, Guinea-Bissau’s health care system is very much dependent on external aid, which makes strong coordination between international donors and the local health authorities critical.

Regardless at what level stillbirths are being addressed, an ongoing objective should be strengthening data collection, both at hospital and community level, with regular feedback to and dialogue with the local clinicians. Ideally, this would include interviews with the women concerning the timeliness of the delivery and the specific circumstances leading to the stillbirth (e.g. when did contractions begin, how did she get to the hospital, was she attended right away, how soon was it decided to do a caesarean section etc.). Active participant observation would likely be of considerable value. Future stillbirth works should also include collection of data on maternal infections and malnutrition. In the community cohort, the BHP currently carries out “verbal autopsy” interviews in relation to childhood deaths, a routine which could potentially be modified to also include stillbirths. Also, research should go into the stigmas related to HIV infection and how this affects pregnancy outcomes such as stillbirth.

## Conclusion

The stillbirth rate at Guinea-Bissau’s principal obstetric hospital is unacceptably high, with most deaths occurring intrapartum (fresh stillbirths). The hospital stillbirth rate exceeded the community stillbirth rate, reflecting selected referrals of high-risk patients, but possibly also sub-standard routines. Antenatal care and maternal education were identified as important focus areas in stillbirth prevention as well. Interventions aimed at reducing the number of stillbirths in Guinea-Bissau, both at hospital and community level, should be of high priority.

## Supporting information

S1 Fig Bissau map. The suburbs in the BHP area (community cohort) are in grey. The HNSM hospital in the city centre is in red.
(PDF)

S2 Fig Stillbirth rate in the community cohort (BHP area) by number of ANC visits attended at local health centres. (PDF)

S3 Fig FSB rate by time of delivery (24-hour clock) at the hospital. Women from outside Bissau city excluded.
(PDF)

S1 Table Characteristics of women from the rest of Bissau vs. community cohort (BHP area) giving birth at the HNSM hospital. (PDF)
