# Correction: Stillbirths in urban Guinea-Bissau: A hospital- and community-based study

**DOI:** 10.1371/journal.pone.0224589

**Published:** 2019-10-24

**Authors:** Morten Bjerregaard-Andersen, Najaaraq Lund, Anne Sofie Pinstrup Joergensen, Frida Starup Jepsen, Holger Werner Unger, Mama Mane, Amabelia Rodrigues, Staffan Bergström, Christine Stabell Benn

The third author’s initials are indexed incorrectly in PubMed. The correct initials are: Joergensen ASP. The fourth author’s initials are indexed incorrectly in PubMed. The correct initials are: Jepsen FS. The fifth author’s initials are indexed incorrectly in PubMed. The correct initials are: Unger HW. The correct citation is: Bjerregaard-Andersen M, Lund N, Joergensen ASP, Jepsen FS, Unger HW, Mane M, et al. (2018) Stillbirths in urban Guinea-Bissau: A hospital- and community-based study. PLoS ONE 13(5): e0197680. https://doi.org/10.1371/journal.pone.0197680.

The ninth author’s initials are indexed incorrectly in PubMed. The correct initials are: Benn CS.
