# Supplementary material for: Stillbirths in urban Guinea-Bissau: A hospital- and community-based study
Source: PLoS One. 2018 May 23;13(5):e0197680. doi: 10.1371/journal.pone.0197680 (PMC5965864; doi:10.1371/journal.pone.0197680)

Supplementary Figure 1: Bissau map. The suburbs in the BHP area (community cohort) are in grey. The HNSM hospital in the city center is in red.

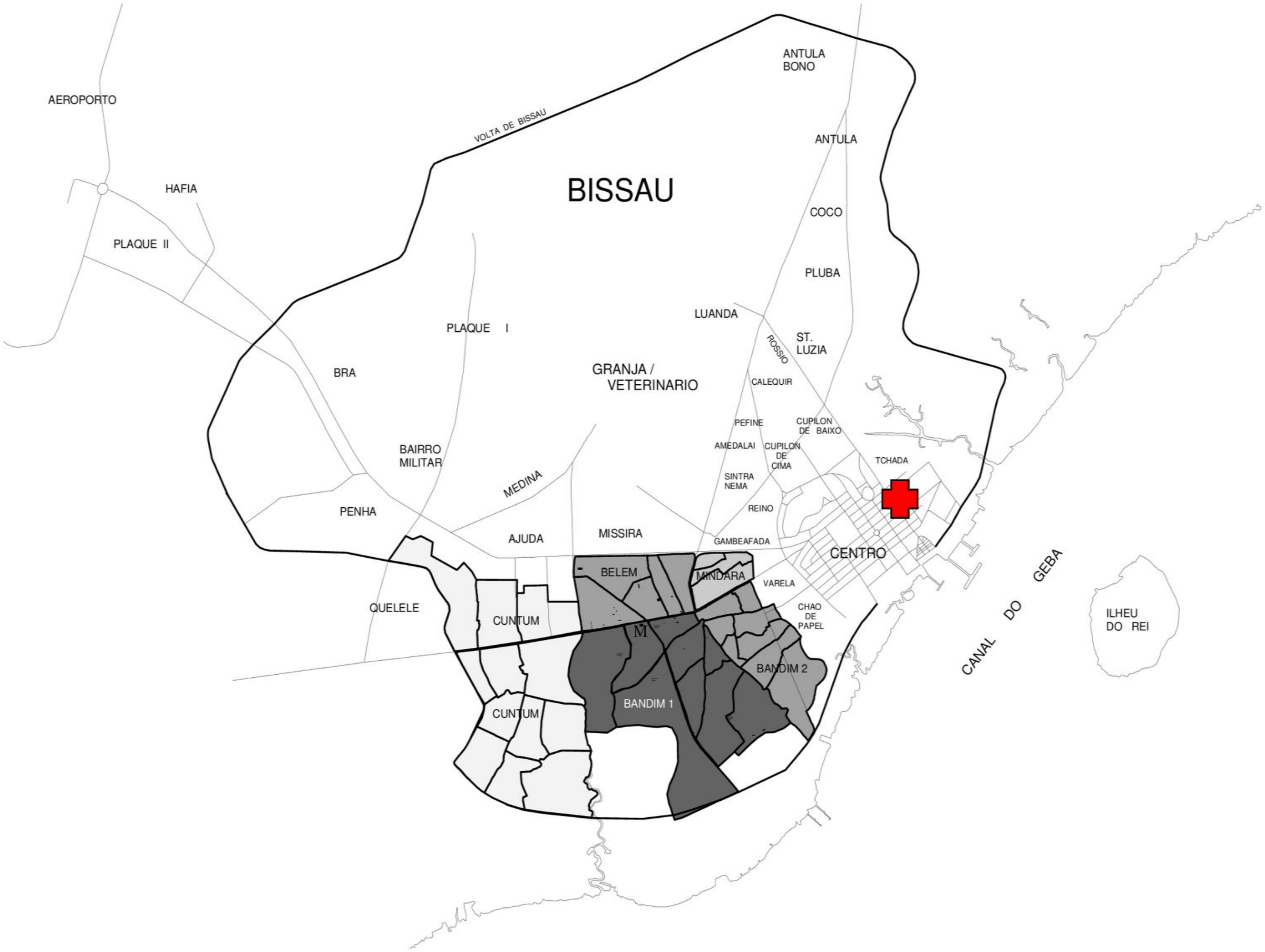

Supplement: S1 Fig — The suburbs in the BHP area (community cohort) are in grey. The HNSM hospital in the city centre is in red. (PDF) [file pone.0197680.s001.pdf]
