# Supplementary material for: Stillbirths in urban Guinea-Bissau: A hospital- and community-based study
Source: PLoS One. 2018 May 23;13(5):e0197680. doi: 10.1371/journal.pone.0197680 (PMC5965864; doi:10.1371/journal.pone.0197680)

Supplementary Figure 2: Stillbirth rate in the community cohort (BHP area) by number of ANC visits attended at local health centres.

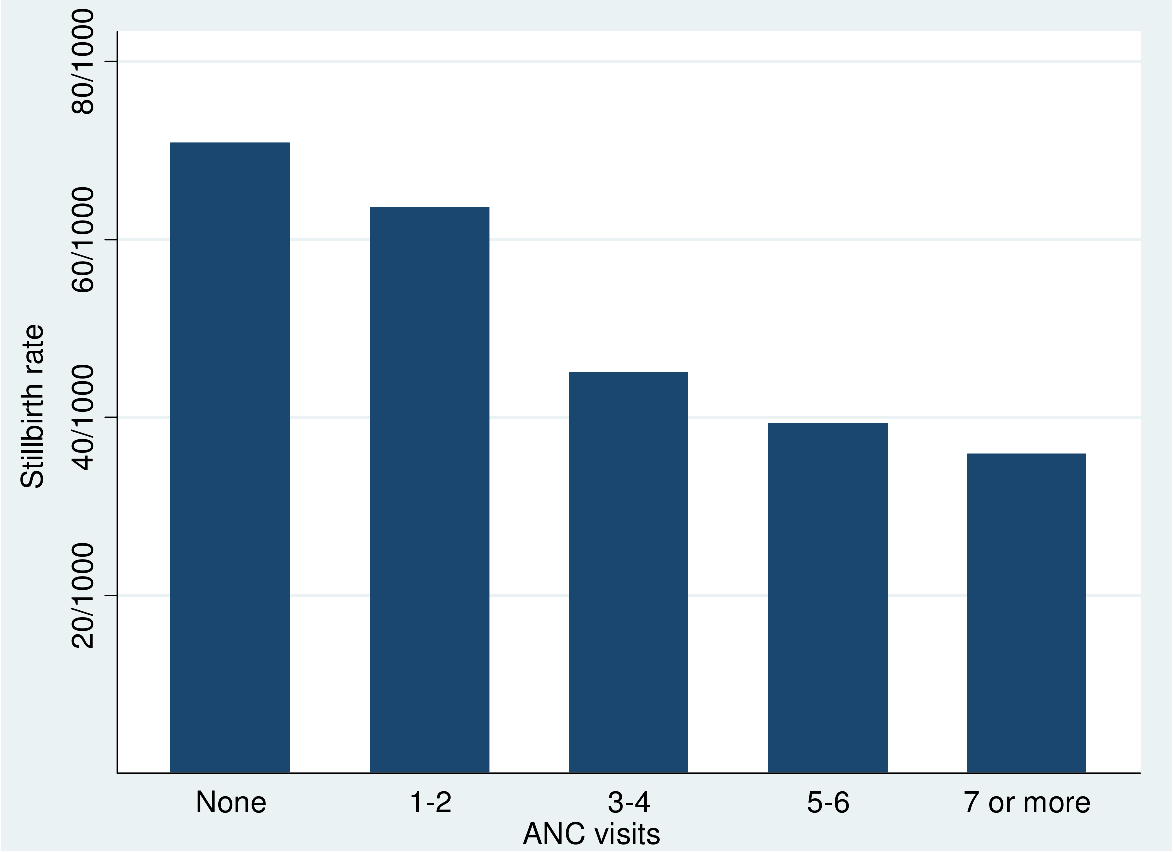

Supplement: S2 Fig — (PDF) [file pone.0197680.s002.pdf]
