# Supplementary material for: Stillbirths in urban Guinea-Bissau: A hospital- and community-based study
Source: PLoS One. 2018 May 23;13(5):e0197680. doi: 10.1371/journal.pone.0197680 (PMC5965864; doi:10.1371/journal.pone.0197680)
Supplement: S3 Fig — Women from outside Bissau city excluded. (PDF) [file pone.0197680.s003.pdf]

Supplementary Figure 3: FSB rate by time of delivery (24-hour clock) at the hospital. Women from outside Bissau city excluded.

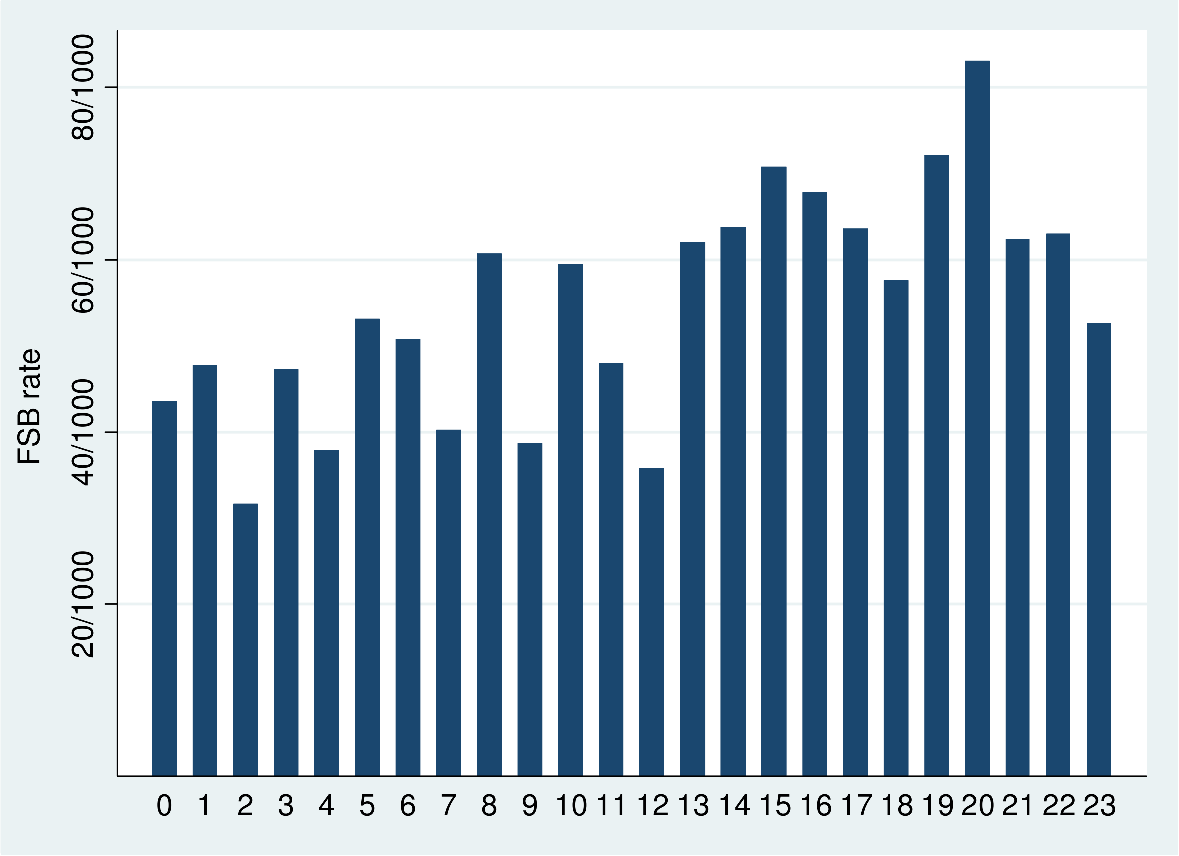

Note: As an example, 2H is 2 a.m. while 20H is 8 p.m.
