# Supplementary material for: Stillbirths in urban Guinea-Bissau: A hospital- and community-based study
Source: PLoS One. 2018 May 23;13(5):e0197680. doi: 10.1371/journal.pone.0197680 (PMC5965864; doi:10.1371/journal.pone.0197680)
Supplement: S1 Table — (PDF) [file pone.0197680.s004.pdf]

Supplementary Table 1: Characteristics of women from rest of Bissau vs. BHP area giving birth at the HNSM hospital

	**Other Bissau** N=27767	**BHP area** N=6723	**P or PR (95% CI)**
Age; years [N=34291]	24.5 (5.80)	24.9 (5.82)	<0.001
Ethnicity; %			
Balanta	24.6 (6843/27767)	8.9 (600/6723)	2.78 (2.54-3.00)
Fula	22.8 (6326/27767)	22.2 (1493/6723)	1.03 (0.97-1.09)
Pepel	11.0 (3055/27767)	23.2 (1560/6723)	0.47 (0.45-0.50)
Mandinga	11.5 (3205/27767)	7.6 (508/6723)	1.53 (1.39-1.68)
Manjaco	6.9 (1914/27767)	12.4 (830/6723)	0.56 (0.51-0.61)
Mancanha	7.5 (2090/27767)	10.0 (674/6723)	0.75 (0.69-0.82)
Other	15.6 (4334/27767)	15.7 (1058/6723)	0.99 (0.93-1.06)
Parity [N=33945]*	2 (1-3)	2 (1-3)	0.35
Married; %	72.0 (19752/27417)	69.6 (4637/6663)	1.04 (1.00-1.07)
Educational level; %			
No schooling	23.5 (6019/25572)	20.5 (1282/6269)	1.15 (1.08-1.22)
1-4 years	17.6 (4503/25572)	15.2 (953/6269)	1.16 (1.08-1.24)
≥5 years	58.9 (15050/25572)	64.3 (4034/6269)	0.91 (0.88-0.95)
HIV infection; %	5.6 (1004/18072)	4.2 (170/4037)	1.32 (1.12-1.55)

Cells are percent (n/N) or mean (SD), except for continuous non-normally distributed data (marked with *) which are presented as median (25%-75% percentiles). For continuous data, we have added the total number [N] of observations.
